# Shifts in *bla* genes and Class 1 integron prevalence in beta-lactamase-producing bacteria before and after the COVID-19 pandemic in Mendoza, Argentina

**DOI:** 10.1128/spectrum.02771-24

**Published:** 2025-07-15

**Authors:** F. Márquez-Friedrichs, M. B. Nolly, A. Ferreyra, L. Zuloaga, S. Dominguez, A. Secotaro, V. S. Rathour, M. T. Damiani, L. Contreras, D. G. Sánchez

**Affiliations:** 1 Laboratorio de Bioquímica e Inmunidad, Instituto de Bioquímica y Biotecnología, Facultad de Ciencias Médicas, Universidad Nacional de Cuyo 28218, Mendoza, Argentina; 2 IMBECU-CONICET, Mendoza, Argentina; 3 Laboratorio de Bacteriología del Hospital Central 502104 , Mendoza, Argentina; 4 Área de Microbiología, Facultad de Ciencias Médicas, Universidad Nacional de Cuyo 28218, Mendoza, Argentina; 5 Laboratorio de Biotecnología, Facultad de Ciencias Aplicadas a la Industria, Universidad Nacional de Cuyo (UNCuyo) 28218, San Rafael, Argentina; 6 Instituto de Ingeniería y Ciencias Aplicadas a la Industria (ICAI), Consejo Nacional de Investigaciones Científicas y Técnicas (CONICET)-UNCuyo, San Rafael, Argentina; JMI Laboratories, North Liberty, lowa, USA

**Keywords:** extended-spectrum beta-lactamases (ESBL), genes coding for ESBL, Class 1 integron, surveillance studies

## Abstract

This study analyzes the molecular epidemiology of *bla* genes and Class 1 integron in broad-spectrum beta-lactamase (BSBL) and extended-spectrum beta-lactamase (ESBL) producing strains of bacteria isolated from clinical samples of hospitalized and ambulatory patients before and after the COVID-19 pandemic. Isolates obtained in two periods were compared: the first corresponding to the years November 2019–March 2020, and the second to the years November 2021–April 2022. We evaluate changes in resistance patterns of antibiotics associated with pressures on the healthcare system and social lockdowns. A total of 156 isolates were analyzed: 78 from the first period (61 hospitalized, 17 ambulatory) and 78 from the second period (47 hospitalized, 31 ambulatory). *Escherichia coli* and *Klebsiella pneumoniae* were the predominant bacterial species, representing 85% of the isolates in both periods. The frequency of ambulatory ESBL-producing isolates increased significantly, from 22% (17/78) to 40% (31/78; *P* < 0.01) in the second period. The prevalence of *bla*_SHV_ increased from 24% (19/78) to 72% (56/78; *P* < 0.01) in the second period, while the *bla*_CTX-M-2_ group, absent in the first period, was detected in 43% (34/78) of isolates from the second period. Strains from the second period exhibited greater genetic complexity, with an increased prevalence of combinations involving three or more *bla* genes, including isolates carrying up to five of such genes. Class 1 integron showed a strong correlation with resistance to ciprofloxacin and trimethoprim-sulfamethoxazole. The gene *bla*_OXA-1_, previously associated with resistance to beta-lactamase inhibitors, did not show a clear pattern in the second period.

**IMPORTANCE:**

Antimicrobial resistance associated with the production of extended-spectrum beta-lactamase (ESBL) represents a critical global health challenge, particularly due to the limited development of new antibiotics. This is the first report from Argentina’s central-west region examining the prevalence of beta-lactamase-encoding genes, providing a framework for future research. Our findings reveal a significant increase in bacteria with the ESBL phenotype, particularly among ambulatory populations post-pandemic, suggesting a concerning spread of multidrug-resistant bacteria outside hospital environments. This could compromise empirical antibiotic treatments for ambulatory patients, increasing the risk of severe complications. Our results highlight the urgent need for ongoing surveillance to detect virulent strains before clonal spread or horizontal gene transfer occurs in the community. They also emphasize the importance of strategies to ensure the prudent use of antimicrobials and mitigate the increasing prevalence of resistance genes, which threatens the effectiveness of current therapeutic options.

## INTRODUCTION

Clinically relevant bacteria exhibit resistance to a variety of antibiotics (ATB), including third and fourth-generation cephalosporins, as well as carbapenems. Bacteria with extended-spectrum beta-lactamases (ESBLs) exhibit resistance to ATB like penicillin, aztreonam, and cephalosporins, while remaining susceptible to carbapenems and beta-lactamase inhibitors, such as clavulanic acid, sulbactam, tazobactam, avibactam, relebactam, and vaborbactam. Patients infected with ESBL-producing bacteria face heightened mortality risks when treated empirically with inappropriate beta-lactam ATB (1). For this challenge, carbapenems are often employed against ESBL (2). Unfortunately, the decline in the development of new drugs has led to alarming projections: the United Nations estimates that by the year 2050, bacterial infections could claim the lives of 10 million people (3, 4).

The OXA-1 and OXA-2 groups strongly hydrolyze aminopenicillin and ureidopenicillin (e.g., piperacillin), but they are less effective over broad-spectrum cephalosporin. This specificity earns them the designation of narrow-spectrum beta-lactamases. They display limited inhibition by clavulanic acid, sulbactam, and tazobactam. Their presence is closely linked to resistance against ampicillin/sulbactam and piperacillin/tazobactam (5–7).

Horizontal gene transfer seems to be responsible for the dissemination of ATB resistance genes. Several mechanisms referred to as “mobilome” are responsible for acquiring and dispersing ATB resistance. Horizontal gene transfer occurs through processes such as conjugation involving conjugative transposons and plasmids, transformation, and transduction (8–11).

Genes can go from the chromosome to plasmids and vice versa. They can duplicate or change their surroundings, leading to an increase or decrease in gene expression. Mechanisms responsible for this are insertion sequences (IS), both simple and complex transposons, introns, and integrons (12–17).

An integron is a dynamic genetic element that encodes a site-specific integrase and a combination of genes referred to as gene cassettes. These cassettes lack a promoter sequence and are expressed in an operon-like manner, utilizing an intron’s promoter.

Remarkably, these cassettes can autonomously remove themselves as non-replicative circles and integrate into distinct elements. The origins and mechanisms that give rise to these cassettes, however, remain unclear (18).

Class 1 integrons are characterized by having two conserved DNA sequences at their 5′ and 3′ ends (5′-CS and 3′-CS) with a variable region in between, containing the gene cassettes.

Within these cassettes, an array of ATB resistance genes (ATB genes) can be encoded, encompassing beta-lactams, aminoglycosides, quinolones, macrolides, chloramphenicol, trimethoprim, sulfonamides, rifampicin, lincomycin, and fosfomycin.

Numerous Class 1 integrons are flanked by IS, facilitating their transfer between bacterial plasmids and chromosomes (14, 18, 19). The prevalence of Class 1 integrons ranges from 22% to 55% among the most frequently encountered Gram-negative bacteria in clinical settings (18).

The aim of this study is to assess the prevalence of ESBL-producing bacteria in hospital and ambulatory samples before and after the COVID-19 pandemic, as well as to compare antimicrobial resistance profiles across both periods. The prevalence and frequency of beta-lactamase genes in hospitalized and ambulatory patients will be determined, and the combinations of beta-lactamase genes by bacterial type will be studied across both periods. Additionally, the prevalence of Class 1 integrons and their association with beta-lactamase genes will be evaluated. The relationship between the presence of integrons and resistance to non-beta-lactam antibiotics in ESBL-producing strains will also be investigated. Finally, the impact of the *bla*OXA-1 gene on resistance to beta-lactam/inhibitor combinations will be assessed. An analysis will also be performed to explore how the prevalence of beta-lactamase genes associated with ESBL-producing strains changed during the COVID-19 pandemic.

## MATERIALS AND METHODS

### Sample collection and processing, bacterial isolates, and antimicrobial susceptibility

A total of 156 bacterial isolates exhibiting the ESBL phenotype were analyzed, equally distributed between two study periods: 78 isolates collected during the prepandemic period, from November 2019 to March 2020, and 78 isolates obtained during the postpandemic period, from November 2021 to April 2022. The bacterial strains were isolated from both ambulatory and hospitalized patients at Mendoza’s Central Hospital. These isolates were further evaluated for the prevalence of resistance genes, specifically those encoding broad-spectrum beta-lactamase (BSBL) and ESBL. We divided samples depending on the patient’s ambulatory or hospitalized origin. Clinical isolates from urine samples, respiratory secretions, soft tissues, and blood were studied. The samples were obtained consecutively, with the only inclusion criterion being that the isolate exhibited an ESBL phenotype, confirmed through phenotypic resistance tests to beta-lactams. Therefore, the analyzed cohort consists exclusively of consecutive isolates of ESBL-producing Gram-negative bacilli from hospitalized and ambulatory patients until the total number of samples to be analyzed was reached. Sample selection and identification were performed consistently across both periods, with no changes in inclusion criteria or diagnostic methodology. No biases were detected in sample collection that could account for the difference in proportions, nor were there changes in access to ambulatory healthcare, the availability of microbiological tests, or hospitalization policies. The bacteria were grown on Tryptone Soy Agar medium (Britania Lab) and then incubated for 18 to 24 hours at 37°C. The bacterial isolates were identified using matrix-assisted laser desorption/ionization time-of-flight mass spectrometry equipment (Becton, Dickinson-Bruker Daltonics Biotyper, USA).

Antimicrobial susceptibility testing and phenotypic confirmation of ESBL production were performed using the BD Phoenix M50 automated system with the integrated BDXpert expert system, which applies interpretation rules established by the Clinical and Laboratory Standards Institute (CLSI). A bacterial suspension was prepared in Phoenix ID broth SP1 and adjusted to 0.5–0.6 McFarland standard using the Phoenix Spec nephelometer (BD). Subsequently, 25 µL of the suspension was added to Phoenix AST broth SP supplemented with the AST Phoenix redox indicator (Alamar Blue-based). The NMIC-406 and NMIC-407 panels, validated for detecting resistance mechanisms including ESBL production through the comparison of third-generation cephalosporins with and without clavulanic acid, were inoculated and incubated in the instrument for automated reading.

Minimum inhibitory concentration (MIC) values and categorical interpretations (susceptible, intermediate, or resistant) were determined according to current CLSI breakpoints. For this study, interpretive criteria from CLSI M100 were applied. Results were managed and interpreted using BD EpiCenter software, version 7.70B.

Additionally, a confirmatory phenotypic test for ESBL detection was conducted using the disk diffusion method. A bacterial suspension adjusted to 0.5 McFarland standard in sterile saline was inoculated onto Mueller-Hinton agar (Britania Lab). Disks containing ceftazidime (30 µg), amoxicillin/clavulanic acid (20/10 µg), cefotaxime (30 µg), and/or ceftriaxone (30 µg) (all from BD) were strategically placed following the recommendations of the WHONET Argentina network and Instituto Nacional de Enfermedades Infecciosas (INEI)—ANLIS “Dr. Carlos G. Malbrán” (20). Plates were incubated at 37°C for 18–24 hours. An increase in the inhibition zone of third-generation cephalosporins toward the amoxicillin/clavulanic acid disk was interpreted as indicative of ESBL production.

The BD products used in this study and their corresponding manuals are available at https://eifu.bd.com/hcp (NMIC-406 Ref: 448869 and NMIC-407 Ref: 448879).

### DNA extraction and preservation from bacteria isolates

Bacterial isolates were grown in a 5 mL Luria-Bertani medium and kept stirring at 37°C overnight. Afterward, the culture was divided as follows: 1.5 mL was used to obtain DNA, and the remaining volume was stored in 30% glycerol at −80°C.

Genomic DNA extraction was performed using the Simgen genomic bacterial DNA column extraction kit following the manufacturer’s recommendations.

### Gene amplification

Once DNA was obtained, we amplified beta-lactamase coding genes and Class 1 integrase (IntI1) coding gene with PCR technique using Taq DNA Polymerase TransStart (Transgene). All primers were synthesized by Eurofins Genomics.

The specific primers used for amplifying BSBL, ESBL, and IntI1 genes are indicated in Table 1.

**TABLE 1:** Set of specific primers for PCR^*a*^

Primer	Gen	Sequence	Product length (pb)
BEL-F	*bla*BEL	GAAACTGCTGCTCTACCCG	788
BEL-R		CGCCTTGCAATTCAGGTGC	
VEB-F	*bla*VEB	CCGATTGCTTTAGCCGTTT	350
VEB-R		GTTCATCGCTGTTGGGGTT	
GES-F	*bla*GES	CGCTTCCATTCACGCACTATT	517
GES-R		TCTCTGAGGTCGCCAGGT	
OXA-1-F	*bla*OXA-1	TACAGCAGCGCCAGTGCA	710
OXA-1-R		TCGACCCCAAGTTTCCTGT	
OXA-2-F	*bla*OXA-2	TTTTCGATGGGACGGCG	385
OXA-2-R		TCCTACCCACCAACCCAT	
OXA-10-F	*bla*OXA-10	CCGAAGCCGTCAATGGTG	571
OXA-10-R		CCAACCCACCATGCGACA	
PER-1-F	*bla*PER	GCCTCACGATCTGGAACC	642
PER-1-R		GCCGTCCATCAGGCAACA	
SHV-F	*bla*SHV	GCGTTATATTCGCCTGTG	757
SHV-R		GCGCTCTGCTTTGTTATTC	
TEM-F	*bla*TEM	GAGTATTCAACATTTCCGTGT	849
TEM-R		AATCAGTGAGGCACCTATC	
CTX-M-group 1-F	*bla*CTX*-M-group 1*	AAAGTGATGGCCGTGGCC	522
CTX-M-group 2-F	*bla*CTX*-M-group 2*	ATGATGACTCAGAGCATTCGC	749
CTX-M-group 1+2-R		GATATCGTTGGTGGTGCCA	
Int1-F	*Intl 1*	CCTCCCGCACGATGATC	280
Int1-R		TCCACGCATCGTCAGGC	

^*a*^ F, the forward primer for amplification; R, the reverse primer for amplification. Primers sequence from Laudy et al. (21).

### Amplified sequence detection

The obtained products were visualized through electrophoresis using a 1% agarose gel in 1× TAE (Tris acetate 0.4 M pH 8.3, EDTA 0.01 M). To estimate PCR product size, a molecular marker (GeneRuler 1 kb Plus DNA Ladder, Invitrogen) was used. Afterward, bands were visualized under blue light on a transilluminator.

### Statistic analysis

Chi-squared distribution analysis was conducted using InfoStat 2020 software, considering *P* < 0.05 of statistical significance (22).

## RESULTS

### Comparison of the prevalence of BSBL and ESBL-producing bacteria in hospital and ambulatory samples: pre-pandemic vs post-pandemic analysis

We analyzed bacterial isolates with an ESBL phenotype from hospitalized and ambulatory patients. A comparison of the collected data was conducted to evaluate the prevalence of the bacteria in these isolates of both groups before and after the pandemic. A total of 78 samples were analyzed in each period. The pre-pandemic period consisted of a total of 61 hospital samples and 17 ambulatory samples. In the post-pandemic period, 47 hospital samples and 31 ambulatory samples were examined.

**Fig 1:**
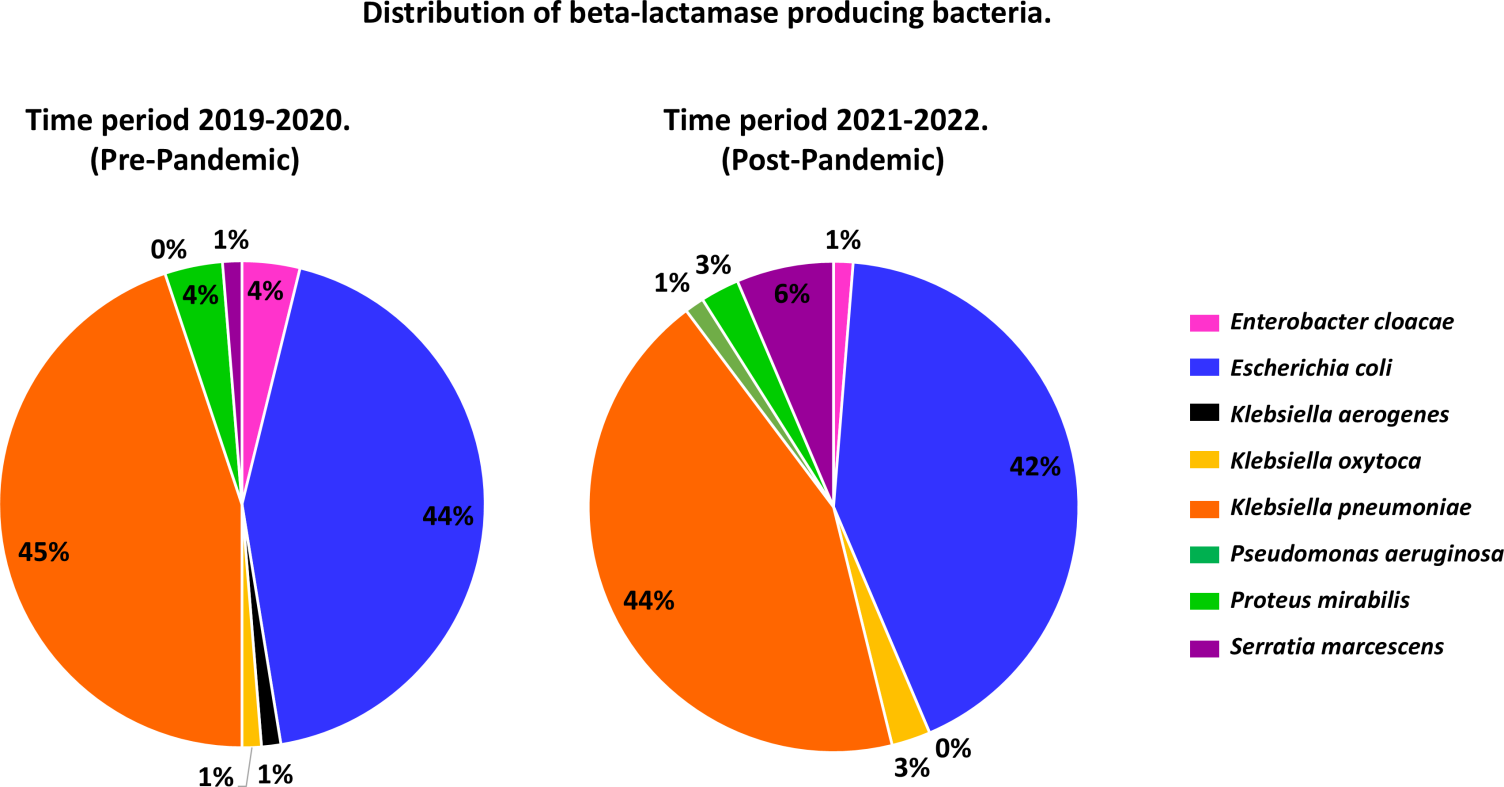
Temporal distribution of ESBL-producing bacteria during the years 2019–2020 and 2021–2022.

The results showed that the most prevalent bacteria in both periods were *Klebsiella pneumoniae* (*K. pneumoniae*) with an approximate prevalence of 44% (34/78 in the pre-pandemic period vs 35/78 in the post-pandemic period). This bacterium was followed by *Escherichia coli* (*E. coli*) with approximately 43% (*n* = 34/78 pre-pandemic vs 33/78 post-pandemic). Other isolated species accounted for approximately 13% of the total number of samples (*n* = 10/78 pre-pandemic vs *n* = 11/78 post-pandemic), including *Serratia marcescens*, *Klebsiella oxytoca*, *Enterobacter cloacae,* and *Proteus mirabilis* among others (Fig. 1).

The antimicrobial resistance profiles of the bacteria from both periods are detailed in Table 2.

**TABLE 2:** Phenotypic antimicrobial-resistance profile of ESBL-producing isolates across both time periods^*a*^

	Period 2019–2020									Period 2021–2022								
Antibiotic	Sensitive		Intermediate		Resistant		Undetermined		Total	Sensitive		Intermediate		Resistant		Undetermined		Total
	AF	RF (%)	AF	RF (%)	AF	RF (%)	AF	RF (%)	AF	AF	RF (%)	AF	RF (%)	AF	RF (%)	AF	RF (%)	AF
SMZ-TMP	9	12	0	0	57	73	12	15	78	21	27	1	1	48	62	8	10	78
Piperacillin-Tazobactam	40	51	4	5	25	32	9	12	78	22	28	3	4	29	37	24	31	78
Meropenem	60	77	1	1	0	0	17	22	78	46	59	1	1	0	0	31	40	78
Imipenem	59	76	0	0	0	0	19	24	78	44	56	0	0	0	0	34	44	78
Ertapenem	59	76	3	4	2	3	14	18	78	54	69	1	1	1	1	22	28	78
Ciprofloxacin	10	13	4	5	60	77	4	5	78	21	27	3	4	50	64	4	5	78
Ceftrioxone	0	0	0	0	65	83	13	17	78	0	0	0	0	56	72	22	28	78
Ceftazidime	8	10	2	3	57	73	11	14	78	1	1	0	0	59	76	18	23	78
Cefepime	1	1	6	8	55	71	16	21	78	0	0	1	1	55	71	22	28	78
Ampicillin-Sulbactam	17	22	1	1	50	64	10	13	78	18	23	3	4	55	71	2	3	78
Amikacin	59	76	1	1	4	5	14	18	78	42	54	3	4	5	6	28	36	78
Gentamicin	43	55	2	3	26	33	7	9	78	35	45	1	1	24	31	18	23	78

^*a*^ The number inside the bars corresponds to the bacterial amount. AF, absolute frequency; RF, relative frequency.

**Fig 2:**
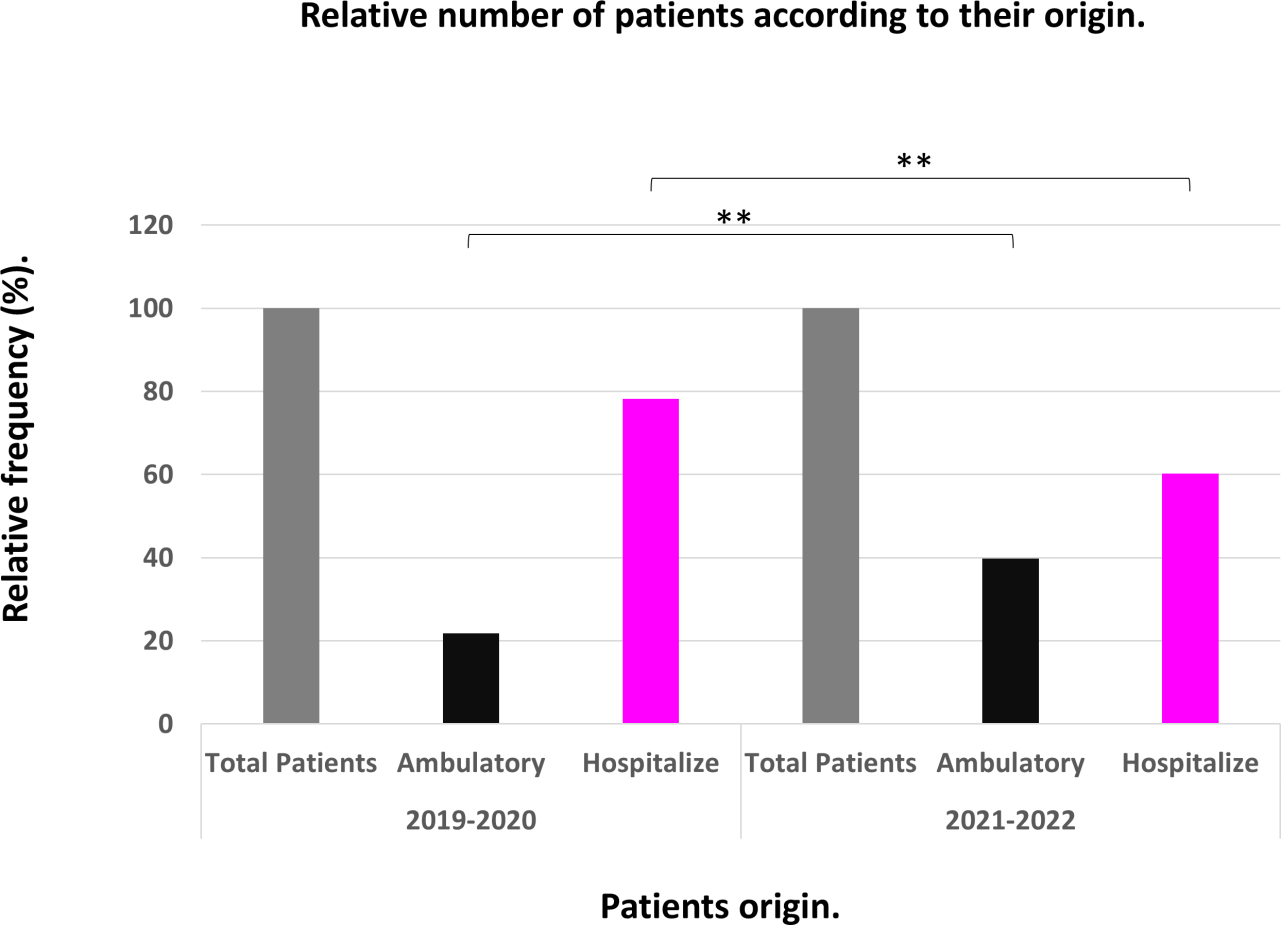
Patient distribution according to their origin during the years 2019–2020 and 2021–2022. **P < 0.01, test independence Chi-squared distribution.

An unexpected finding was the increase in the number of ambulatory samples in the post-pandemic period by approximately 82% compared to the pre-pandemic period, *P* < 0.01 (Fig. 2).

### Analysis of the prevalence of beta-lactamase genes in ambulatory and hospitalized patients: pre-pandemic vs post-pandemic changes

We assessed the frequency of genes encoding beta-lactamases in isolates from ambulatory and hospitalized patients. During the pre-pandemic period, 11 beta-lactamase genes were analyzed: *bla*BEL, *bla*VEB, *bla*GES, *bla*OXA-1, *bla*OXA-2, *bla*OXA-10, *bla*PER-1, *bla*SHV, *bla*TEM, *bla*CTX-M-group 1, and *bla*CTX-M-group 2. The most prevalent genes were *bla*OXA-1, *bla*CTX-M-group 1, *bla*SHV, and *bla*TEM, while no strains expressing the other genes were identified. For this reason, in the post-pandemic period, the focus was placed on the most relevant genes from the previous period, including *bla*CTX-M-2, due to its high prevalence in Latin America.

**Fig 3:**
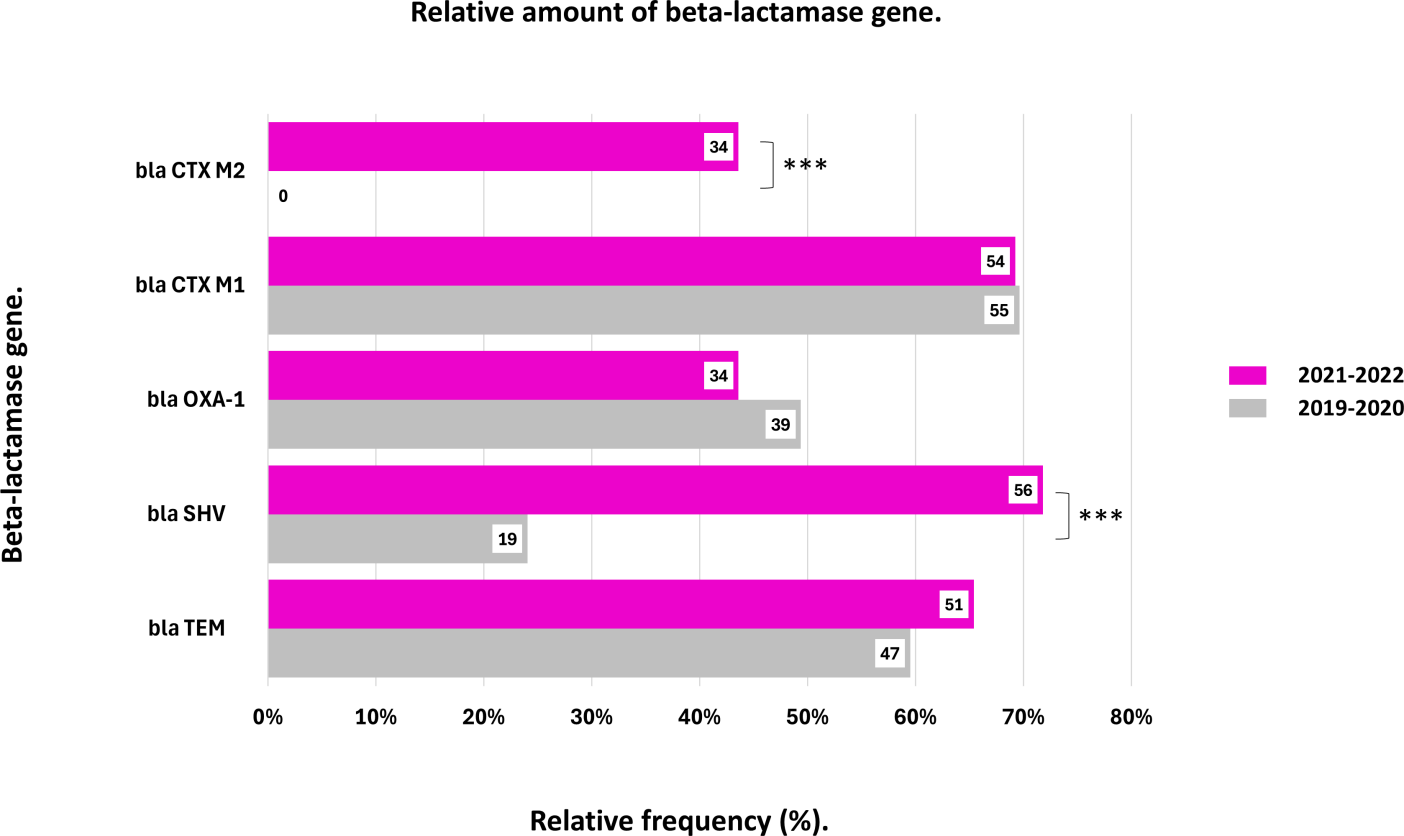
Frequency of beta-lactamase genes during the years 2019–2020 and 2021–2022. ***P < 0.01, test of independence Chi-squared distribution.

Figure 3 illustrates the prevalence of these genes before and after the pandemic. A significant increase in the prevalence of *bla*CTX-M-group 2 and *bla*SHV was observed (*P* < 0.01). Specifically, the prevalence of *bla*CTX-M-group 2 increased from 0% (*n* = 0/78) during the pre-pandemic period to 43% (*n* = 34/78) in the post-pandemic period. For *bla*SHV, its prevalence increased from 24% (*n* = 19/78) pre-pandemic to 72% (*n* = 56/78) post-pandemic. *bla*TEM showed a tendency to increase in the post-pandemic period. While the other genes, *bla*OXA-1 and *bla*CTX-M-group 1, showed a tendency to decrease in the post-pandemic period.

**Fig 4:**
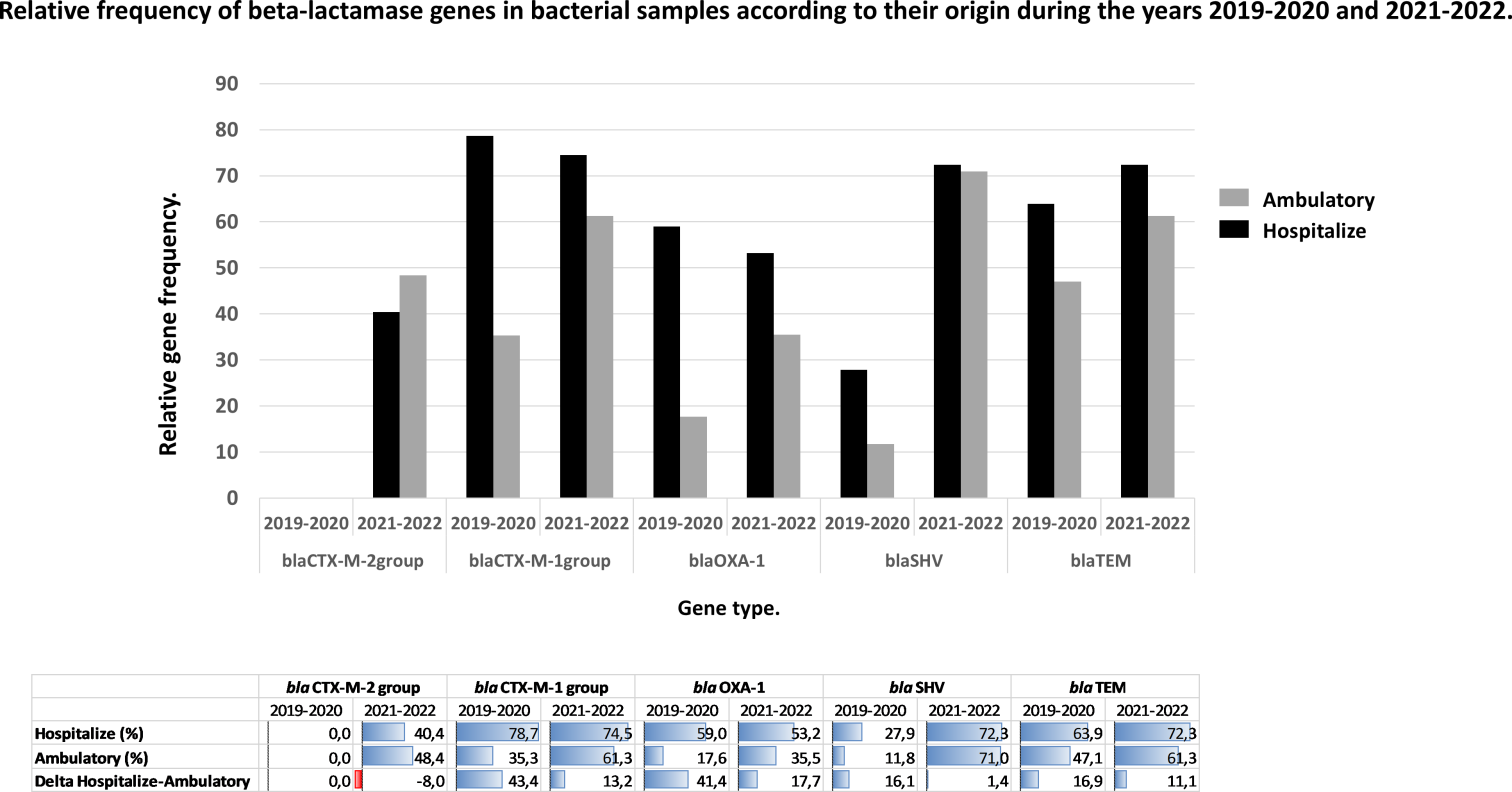
Frequency of beta-lactamase genes in hospitalized and ambulatory patients over two time periods. Delta refers to the difference between hospitalized and ambulatory patients.

Next, we analyze the relative frequency of beta-lactamase genes in bacterial samples, differentiating between hospitalized and ambulatory patients during the pre- and post-pandemic periods (Fig. 4).

The *bla*CTX-M-group 2 gene, absent during the pre-pandemic period, emerged during the SARS-CoV-2 pandemic, reaching both populations: hospitalized 40.43% (*n* = 19/47) and ambulatory 48.39% (*n* = 15/31).

Notably, the prevalence of the *bla*SHV gene not only demonstrated a significant increase in the post-pandemic period but also exhibited a more homogeneous distribution between hospital and ambulatory patient samples.

The prevalence of *bla*SHV in the pre-pandemic period was 27.87% (*n* = 17/61) in hospital samples and 11.76% (*n* = 2/17) in ambulatory samples. In the post-pandemic period, the prevalence of *bla*SHV increased, reaching approximately 70.97% in both hospital (*n* = 34/47) and ambulatory (*n* = 22/31) samples.

For the remaining genes, a more balanced distribution between hospital and ambulatory settings is becoming evident in the post-pandemic period. For instance, the relative frequency delta of the *bla*CTX-M-group 1 gene decreased from 43% in the pre-pandemic period to 13% in the post-pandemic period between hospital and ambulatory settings, driven by an increase in the prevalence of this gene in ambulatory samples during the post-pandemic phase (Fig. 4).

### Analysis of resistance genes by bacterial type and sample origin: pre-pandemic and post-pandemic comparison

The relative frequency of beta-lactamase genes was analyzed based on the origin of the samples, classifying them as either hospital or ambulatory, during the pre-pandemic and post-pandemic periods (data not shown). The most relevant differences observed were as follows. The *blaSHV* gene showed an increase in the post-pandemic period in both hospitalized and ambulatory patients, with the most significant increase observed in *E. coli*, where the frequency rose 6.6-fold in both categories. For *K. pneumoniae*, the frequency increased twofold in both hospitalized and ambulatory samples.

On the other hand, the *blaCTX-M-1* group experienced a 17% decrease in *K. pneumoniae* hospital samples during the post-pandemic period, while the frequency approximately doubled in ambulatory samples, both for *K. pneumoniae* and *E. coli*. Similarly, the *blaOXA-1* gene followed a similar trend to that of the *blaCTX-M-1* group, showing a 23% decrease in *K. pneumoniae* hospital samples during the post-pandemic period. A significant increase in ambulatory samples, with a 129% rise in *E. coli* and a 67% rise in *K. pneumoniae* for the *blaOXA-1* gene during the post-pandemic period.

### Analysis of the prevalence of beta-lactamase gene combinations in bacteria from hospitalized and ambulatory patients during pre-pandemic and post-pandemic periods

**Fig 5:**
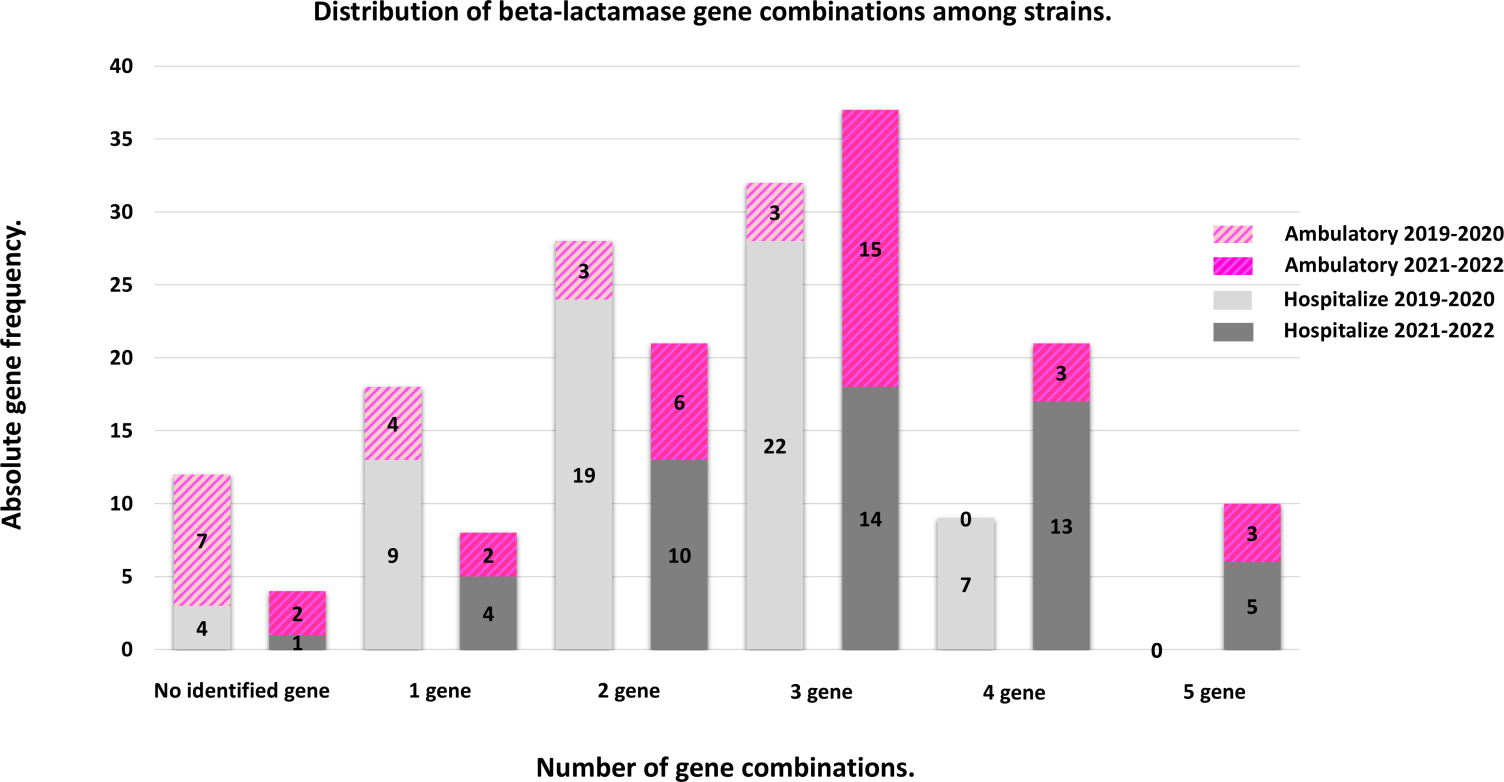
Frequencies of beta-lactamase gene combination distributions among strains isolated from hospitalized and ambulatory patients during the years 2019–2020 and 2021–2022.

During both the pre-pandemic and post-pandemic periods, combinations of two and three beta-lactamase genes were predominant, particularly in hospitalized patients. However, in the post-pandemic period, a significant increase in the prevalence of bacteria harboring three or more genes was observed, with a trend toward the emergence of strains containing four or five genes. Unlike the pre-pandemic period, during the post-pandemic period, hospital isolates with five genes and ambulatory isolates with four and five genes were identified. In addition, we observed that phenotypically ESBL-producing bacteria with none of the studied genes detected were more common in samples of both hospitalized and ambulatory patients from the pre-pandemic period (Fig. 5).

Beta-lactam resistance gene combinations were analyzed in relation to the origin and time frame of sample collection. The results are summarized in Table 3.

**TABLE 3:** Frequency of identified beta-lactamase genes in bacterial isolates during the years 2019–2020 and 2021–2022

		Ambulatory		Hospitalized		
	Genes de resistencia	2019–2020	2021–2022	2019–2020	2021–2022	Total
1 gene	blaOXA-1	0	0	1	0	1
	blaCTX M1	0	1	3	2	6
	blaCTX M2	0	0	0	0	0
	blaSHV	1	1	0	1	3
	blaTEM	3	0	5	1	9
2 gene	bla TEM + bla CTX M1	2	2	7	2	13
	bla TEM + bla SHV	0	2	0	2	4
	bla TEM + bla OXA-1	0	0	2	0	2
	bla TEM + bla CTX M2	0	0	0	2	2
	bla OXA-1 + bla CTX M1	1	0	9	0	10
	bla SHV + bla CTX M1	0	1	1	2	4
	bla SHV + bla OXA-1	0	1	0	1	2
	bla SHV + bla CTX M2	0	0	0	1	1
3 gene	bla OXA-1 + bla CTX M1 + bla CTX M2	0	1	0	2	3
	bla OXA-1 + bla CTX M1 + bla SHV	0	1	3	2	6
	bla OXA-1 + bla CTX M1 + bla TEM	2	0	13	1	16
	bla OXA-1 + bla CTX M2 + bla TEM	0	1	0	0	1
	bla OXA-1 + bla TEM + bla SHV	0	1	1	0	2
	bla CTX M1 + bla CTX M2 + bla SHV	0	2	0	1	3
	bla CTX M1 + bla CTX M2 + bla TEM	0	2	0	0	2
	bla CTX M1 + bla TEM + bla SHV	1	3	5	7	16
	bla CTX M2 + bla TEM + bla SHV	0	4	0	1	5
4 gene	bla OXA-1 + bla CTX M1 + bla CTX M2 + bla SHV	0	2	0	2	4
	bla CTX M1 + bla CTX M2 + bla TEM + bla SHV	0	0	0	1	1
	bla CTX M2 + bla OXA-1 + bla TEM + bla SHV	0	0	0	2	2
	bla CTX M1 + bla OXA-1 + bla TEM + bla SHV	0	1	7	6	14
	bla OXA-1 + bla CTX M1 + bla CTX M2 + bla TEM	0	0	0	2	2
5 gene	bla OXA-1 + bla CTX M1 + bla CTX M2 + bla TEM + bla SHV	0	3	0	5	8
0 gene	No identified beta-lactamase gene	7	2	4	1	14
Total		17	31	61	47	156

### Class I integron: comparison between pre-pandemic and post-pandemic periods

#### Distribution of Class I integron in ESBL-phenotype samples according to patients’ origin

**Fig 6:**
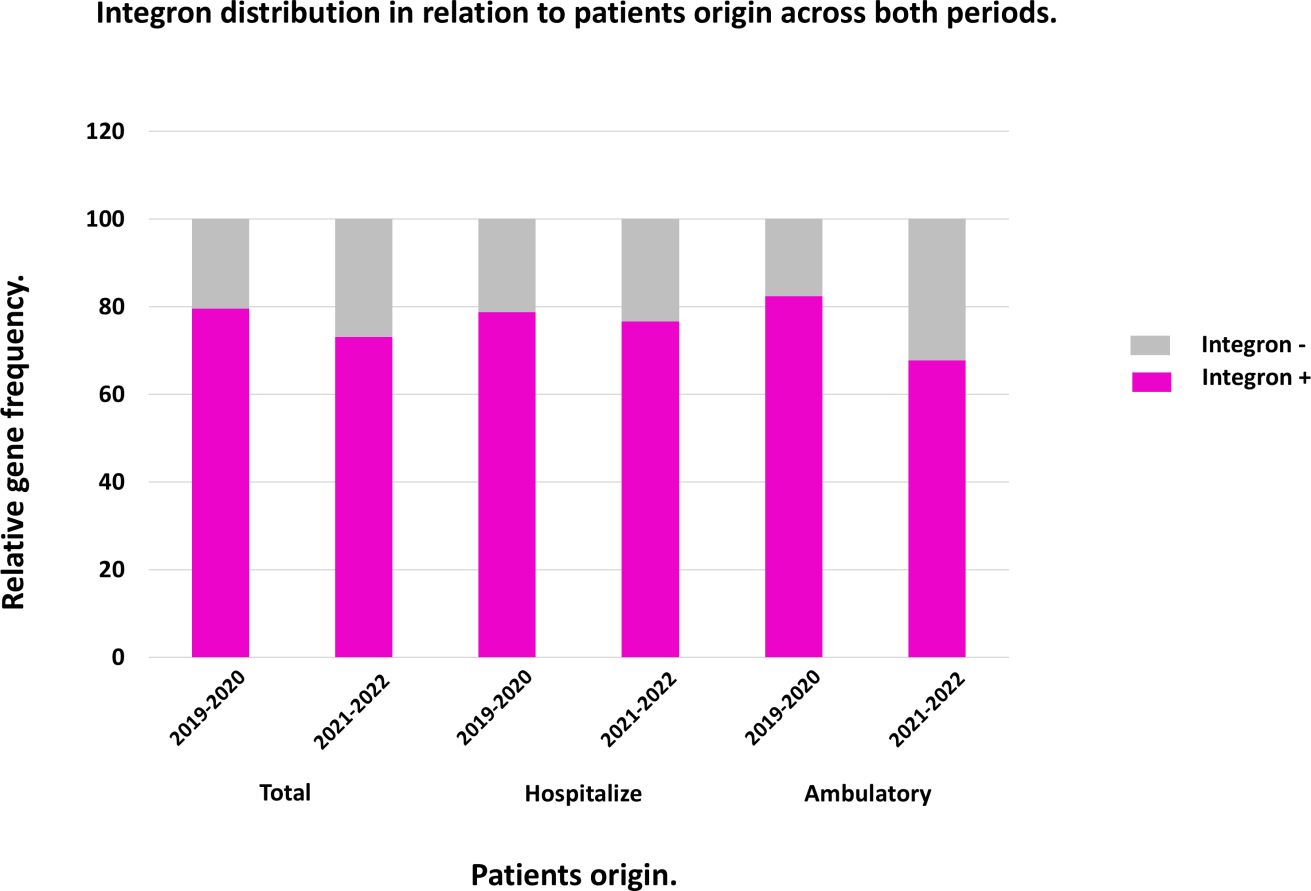
Integron distribution among strains from hospitalized and ambulatory patients during the years 2019–2020 and 2021–2022.

The prevalence of Class I integron was determined by amplifying the conserved sequence of the integrase gene (Intl) by PCR. The results indicate that integrons were frequently detected in BSBL and ESBL-producing bacterial samples. In the total amount of samples, a slight decrease (approximately 6%) in Class I integron prevalence was observed in the post-pandemic period. In hospital samples, the reduction was minimal (2%). While in ambulatory samples, the decrease was more pronounced, reaching 14% (Fig. 6).

Regarding Class I integron prevalence by bacterial species, the results were similar across both periods. In *E. coli*, the prevalence was approximately 71% (24/34 pre-pandemic and 24/33 post-pandemic). In *K. pneumoniae*, integron prevalence decreased significantly from 91% (32/35) in the pre-pandemic period to 73% (27/34) in the post-pandemic period. This reduction was observed in isolates from both ambulatory and hospitalized patients (100% vs 75% and 90% vs 80%, respectively).

#### Evaluation of Class I integron prevalence and its relationship with the number of beta-lactamase genes in ESBL strains

**Fig 7:**
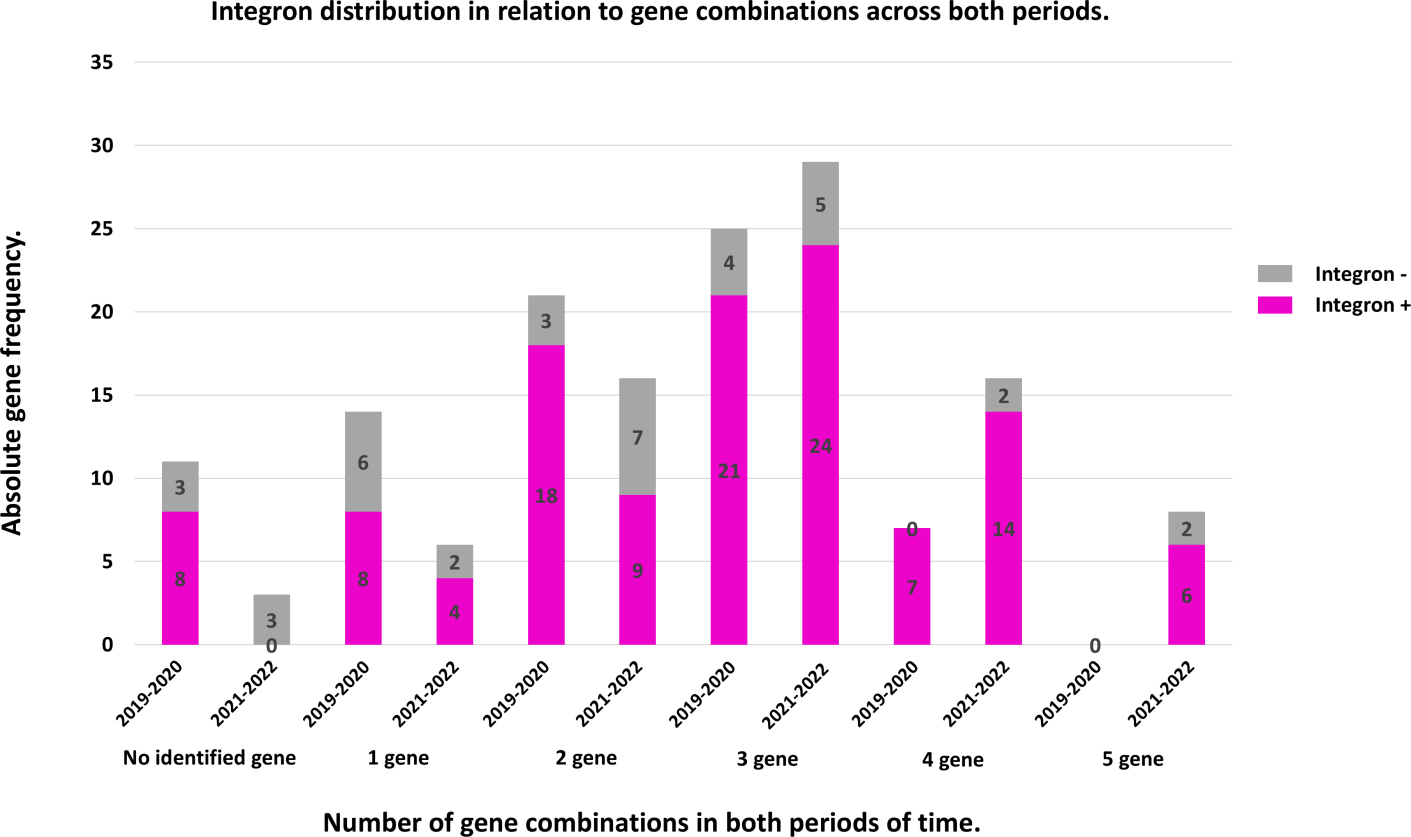
Integron distribution in relation to gene combination during the years 2019–2020 and 2021–2022.

Since integrons have the capacity to accumulate genes, we evaluated their prevalence in relation to the number of beta-lactamase genes present in each strain. The analysis of the distribution of strains with Class I integron based on the number of identified genes between the pre-pandemic and post-pandemic periods revealed interesting patterns (Fig. 7).

In the group of strains with a single gene, the proportion of strains with and without integrons was almost equally distributed in both periods. In the pre-pandemic period, 53% of strains had integrons while 47% did not. In the post-pandemic period, a similar distribution was maintained, with 57% of strains having integrons and 43% without.

As the number of identified genes increases, the proportion of strains with integrons begins to rise. In the group of strains with two genes, 85% had integrons in the pre-pandemic period, persisting this trend in the post-pandemic period, with 75% of strains having integrons.

The tendency becomes more pronounced in strains with three genes, where 84% of strains had integrons in the pre-pandemic period, and 85% of strains had integrons in the post-pandemic period. This increase suggests that strains with integrons become more prevalent among those with multiple genes.

In the groups with four and five genes, all strains had integrons. In the group of strains with four genes, in the pre-pandemic period, seven strains with integrons and no strains lacking integrons were identified. In the group of strains with five genes, in the post-pandemic period, all strains had integrons. This suggests that the presence of integrons is strongly associated with a higher number of beta-lactamase genes, indicating a trend toward increased genetic complexity in these strains.

### Relationship between integrons and non-beta-lactam antibiotic resistance in BSBL and ESBL-producing strains

It has been reported that BSBL and ESBL-producing isolates and those harboring Class 1 integrons often exhibit resistance to non-beta-lactam antibiotics, particularly Ciprofloxacin and Trimethoprim-Sulfamethoxazole. In this context, we aimed to determine if this correlation applies to the ESBL isolates analyzed in this study.

Our findings, consistent with previous studies, indicate that resistance to Ciprofloxacin and Trimethoprim-Sulfamethoxazole correlates with the presence of Class 1 integrons (*P* < 0.001 in both antibiotics). Ciprofloxacin resistance was analyzed in a total of 74 strains from both periods, while Trimethoprim-Sulfamethoxazole resistance was evaluated in 66 strains during the pre-pandemic period and 70 strains in the post-pandemic period.

During the pre-pandemic period, the percentage of strains resistant to these antibiotics was approximately 86% (64/74 for Ciprofloxacin and 57/66 for Trimethoprim-Sulfamethoxazole). In the post-pandemic period, resistance rates decreased, with 72% (53/74) of strains resistant to Ciprofloxacin and 70% resistant to Trimethoprim-Sulfamethoxazole (49/70).

Although integron prevalence in strains from both periods was similar, with a slight decrease in the post-pandemic period (78% pre-pandemic and 73% post-pandemic) (Fig. 6), the relationship between resistant and sensitive strains, with or without integrons, varied significantly across both periods for both groups of antibiotics (data not shown).

In the pre-pandemic period, 91% of strains with integrons were resistant to Ciprofloxacin (51/53), and 96% (50/52) were resistant to Trimethoprim-Sulfamethoxazole. In contrast, among strains without integrons, resistance was 68% (11/16) for Ciprofloxacin and 50% (7/14) for Trimethoprim-Sulfamethoxazole.

In the post-pandemic period, the proportion of resistant strains, both with and without integrons, decreased compared to the pre-pandemic period, for both Ciprofloxacin and Trimethoprim-Sulfamethoxazole. Among strains with integrons, Ciprofloxacin resistance was 80% (43/54), representing a 12% reduction compared to the previous period, while the reduction for Trimethoprim-Sulfamethoxazole was 6%. In strains without integrons, the reduction in resistance was more pronounced: 19% for Ciprofloxacin and 17% for Trimethoprim-Sulfamethoxazole.

### Evaluation of the impact of the blaOXA-1 gene on resistance to beta-lactam/inhibitor combinations

Given recent evidence that the blaOXA-1 gene contributes to beta-lactam resistance in the presence of beta-lactamase inhibitors, we assessed its impact on resistance to Ampicillin-Sulbactam and Piperacillin-Tazobactam (6). In the first period, statistical analysis showed a significant increase in the risk of resistance to Piperacillin-Tazobactam and Ampicillin-Sulbactam in strains carrying the blaOXA-1 gene (*P* < 0.001 and *P* < 0.05, respectively), doubling the risk of resistance by 50%. However, in the second period, the blaOXA-1 gene did not show a significant impact on resistance to these β-lactam/inhibitor combinations (data not shown).

## DISCUSSION

In Argentina, COVID-19 mobility restrictions were implemented in multiple phases with varying levels of intensity, approximately 270 to 300 days in Mendoza. Starting with the Social, Preventive, and Mandatory Isolation in March 2020 until November 2020, the population faced 90 days of strict quarantine. This was followed by a phase of partial easing and then the Social, Preventive, and Mandatory Distancing until March 2021. A second wave of COVID-19 led to new restrictions from April to July 2021, culminating in a gradual reopening and near-total normalization in December 2021. We defined the cutoff points for our study periods as follows: samples collected before March 2020 were classified as part of the pre-pandemic period. From March 2020 to July 2021, a social lockdown was established. After July 2021, we restarted sample collection, classifying these samples as part of the post-pandemic period. This study evaluates the impact of the COVID-19 pandemic on the prevalence of beta-lactamase genes in BSBL and ESBL-producing bacteria isolated from hospitalized and ambulatory patients at Mendoza’s Central Hospital. We analyzed the molecular epidemiology of the genes associated with the ESBL phenotype, investigated the presence of Class 1 integron, and examined the *blaOXA-1* gene, which is linked to increased resistance to penicillin-beta-lactamase inhibitors in BSBL and ESBL-producing bacteria (6).

The most prevalent isolates were *E. coli* and *K. pneumoniae*. In the post-pandemic period, a pronounced increase in the number of ESBL-positive samples from ambulatory patients was observed, with figures approximately double those observed in the pre-pandemic period. The increase in the proportion of samples from ambulatory patients with an ESBL phenotype could be due to a higher attendance of these patients at high-complexity centers after the failure of initial empirical treatments in primary care, often based on beta-lactams, possibly with beta-lactamase inhibitors or cephalosporins. Another factor to consider is that, during the pandemic, the overload on the healthcare system and the limited availability of hospital beds may have led to some patients with ESBL-producing Gram-negative bacilli infections being managed in ambulatory settings, potentially contributing to the spread of these bacteria among non-hospitalized populations. Additionally, the rise in self-medication during the pandemic may have exerted greater selective pressure on bacterial populations in the community, promoting the dissemination of beta-lactam resistance genes. Currently, health policies are being implemented to restrict access to antibiotics without a properly justified prescription, which could impact the epidemiology of ESBL-producing Gram-negative bacilli infections in the community. We consider this type of study essential, as it highlights the need to strengthen controls, especially for antibiotics. A significant rise in the prevalence of *blaSHV* genes and the *bla*CTX-M-2 group was also identified, with both increasing over sixfold in the post-pandemic period. Furthermore, the number of beta-lactamase-encoding genes per strain increased in samples from both ambulatory and hospitalized patients.

Numerous authors have addressed the impact of the pandemic on the rise of multidrug-resistant bacteria (23–27). For example, a report from the Centers for Disease Control and Prevention examines this issue in relation to the increase in antimicrobial-resistant strains (28). Most of these studies suggest that several factors contributed to the rise in resistance, including prolonged hospitalization with the use of catheters and ventilators, reduced medical staff, diminished preventive practices, supply issues, and, to a large extent, an increase in antibiotic use due to inappropriate prescriptions and self-medication (28–31). It is estimated that beta-lactams were the primary class of antibiotics administered during the pandemic period (25, 31, 32).

The prevalence of ESBL genes in Latin America varies by country, with a global trend toward the predominance of *blaCTX-M* (33, 34). In Chile, a country with notable tourist and economic influences on Mendoza, Argentina, it was reported that the most prevalent genes were *blaSHV* (81%) and *blaCTX-M-group 1* (84.7%), followed by *blaTEM* (73%) and *blaCTX-M-group 2* (20%) (35). A multicenter study conducted in central Chile, which analyzed 22 *K*. *pneumoniae* KPC+ strains, found that 100% of the samples carried *blaSHV* and *blaTEM*, while 45% had *blaCTX-M* (18% *blaCTX-M-15* and 27% *blaCTX-M-2*) (36). In Mexico, Colombia, and Brazil, the *blaSHV* gene predominates, particularly *blaSHV-5* and *blaSHV-12*. In Brazil, both *blaCTX-M* (34%) and *blaSHV* (63%) are prevalent. In Bolivia, *blaCTX-M-2* is detected in 71% of cases, and *blaCTX-M-43* in 21% (37). In Argentina, only *blaCTX-M-group 2* genes had been reported until 2002. By 2010, *blaCTX-M-group 2* represented 50% and *blaCTX-M-group 1* represented 40% of the reported cases (38). Additionally, the *blaTEM* gene was detected in 50% of the samples (39). This increase in the prevalence of *blaCTX-M-group 1* aligns with the current global trend, where *blaCTX-M-group 1* is the most widely distributed ESBL genotype (35, 40–43).

In our study, during the period 2019–2020, the most prevalent genes were *blaCTX-M-group 1* at 70% (n: 55/78), followed by *blaTEM* at 65% (51/78), *blaOXA-1* at 50% (39/78), and *blaSHV* at 24% (19/78). Surprisingly, no positive samples for *blaCTX-M-group 2* were found. However, in the post-pandemic period (2021–2022), we observed a significant increase in the prevalence of *blaCTX-M-group 2* (34% vs 0%) and *blaSHV* (72% vs 24%) compared to the previous period.

Regarding isolates exhibiting an ESBL phenotype in the absence of detectable beta-lactamase-encoding genes, 11 such strains were identified in the first period. Of these, three showed resistance to ampicillin/sulbactam, suggesting a possible involvement of plasmid-mediated AmpC enzymes or chromosomal AmpC hyperproduction. In the remaining eight isolates, the phenotype may be attributable to β-lactamase variants not included in this preliminary screening, such as *blaCTX-M-8*, *blaCTX-M-9*, or *blaCTX-M-25* (35), as well as the possible presence of *blaOXA-163*, a variant of *blaOXA-48* reported in Argentina since 2011 (44, 45). This variant displays an atypical biochemical profile, with significant hydrolytic activity against extended-spectrum cephalosporins and limited activity against carbapenems, while remaining susceptible to inhibition by β-lactamase inhibitors such as clavulanic acid, sulbactam, and tazobactam (20, 46, 47). This biochemical behavior may result in phenotypic patterns consistent with class A ESBLs according to the Ambler classification, producing positive results in conventional inhibitor-based phenotypic tests, which may lead to misclassification in the absence of molecular characterization.

In the second period, three additional isolates with this same profile were detected: two *Serratia marcescens* and one *Klebsiella oxytoca*, all resistant to ampicillin/sulbactam. In these species, resistance is typically mediated by chromosomally encoded or plasmid-mediated AmpC beta-lactamases, which may account for the observed phenotypic profile.

Although numerous studies have addressed the rise of multidrug-resistant strains during the pandemic, few compare the specific prevalence of the implicated genes.

Amato et al. (48) conducted a study on *E. coli* strains resistant to third-generation cephalosporins in children from semi-rural communities in Quito, Ecuador. The study analyzed the prevalence of genes using sequencing data from 90 strains in 2018 and 45 strains in 2021. A variation in the prevalence of the CTX-M-1 group was observed, increasing from 52% in 2018 to 62% in 2021, while the prevalence of SHV decreased from 6% (2018) to 2% (2021), and the CTX-M-2 group declined from 2% (2018) to 0% (2021) (48).

A longitudinal study conducted on wastewater effluents from four cities in southwest England between 2020 and 2022 examined the relative abundance of five resistance genes, including *blaCTX-M*, and Class 1 integrons. The gene loads, normalized to 16S rRNA, revealed a statistically significant increase in blaCTX-M, which was closely associated with the abundance of amoxicillin in the community. Additionally, Class 1 integrons, sul1 (associated with sulfonamides), and qnrS (related to quinolones) also showed increased abundance (49).

In our post-pandemic analysis, we observed a trend toward the accumulation of resistance genes per strain in both ambulatory and hospitalized patient samples. Notably, ≥75% of the strains that accumulated three, four, or five resistance genes also carried the Class 1 integron. This could be interpreted as the accumulation of genes within the integron, or that both the integron and the resistance genes, which may not necessarily be related, could coexist in large conjugative plasmids, as demonstrated by the study of Halder et al. (50).

Resistance to ciprofloxacin typically results from chromosomal alterations, primarily through mutations in topoisomerases. However, there are also reports suggesting that it may be associated with the acquisition of resistance genes. Among these genes are qnr, which encodes a protein that protects gyrase and topoisomerase IV from the action of quinolones, and the aac(6′)-Ib-cr gene, which can inactivate ciprofloxacin (51–53). High levels of quinolone and aminoglycoside resistance have been observed in *E. coli* and *K. pneumoniae* strains producing ESBLs in South Korea, a phenomenon notably associated with the presence of the aac(6′)-Ib-cr gene in the analyzed strains (54). Furthermore, it has been observed that many of these genes are linked to Class 1 integrons (55, 56). It is important to note that, in general, the expression of these genes elevates the MIC values, which may not always meet the resistance breakpoint for quinolones. This could facilitate the continued use of these antibiotics, generating selective pressure that favors the emergence of chromosomal mutants and, ultimately, higher levels of resistance (53, 57).

A recent study identified *blaOXA-1* as the mechanism responsible for 24.1% of resistance to piperacillin/tazobactam (58). In line with this, similar observations were reported in Spain, where *blaOXA-1* accounted for 26.1% of the resistance to amoxicillin/clavulanic acid (59). Similar results were obtained in a study conducted in England in 293 isolates with an ESBL phenotype. The prevalence rates for beta-lactamase genes were *blaCTX-M-1* (78.2%), *blaOXA-1* (50.9%), and *blaTEM-1* (variant 191) (46.8%). This study identified a statistical correlation between the prevalence of *blaOXA-1* and susceptibility to penicillin/beta-lactamase inhibitor combinations. The presence of *blaOXA-1* increased the strain’s tolerance to these combinations, resulting in a shift in the MIC. For instance, in the case of piperacillin/tazobactam, the modal MIC increased from 2 mg/L (in the absence of *blaOXA-1*) to 8 or 16 mg/L (in the presence of *blaOXA-1*). Similarly, for amoxicillin/clavulanic acid, the change was from 4 or 8 mg/L (absence of *blaOXA-1*) to 16 mg/L (presence of blaOXA-1) (6). Resistance to penicillin/beta-lactamase inhibitor combinations in strains harboring the *blaOXA-1* gene may be explained by the suboptimal inhibitory activity of commonly employed beta-lactamase inhibitors against this OXA-1 enzyme.

In the samples from the first period of our study, we observed a significant correlation between the presence of the *blaOXA-1* gene and resistance to the ampicillin/sulbactam combination (*P* = 0.001) and piperacillin/tazobactam (*P* = 0.027), in agreement with previous findings (6). However, in the second period, no clear relationship was found between the presence of the *blaOXA-1* gene and resistance to penicillin/beta-lactamase inhibitor combinations. The lack of correlation between *blaOXA-1* and resistance to penicillin/beta-lactamase inhibitor combinations in the second period may be attributed to several factors. For instance, resistance to piperacillin/tazobactam (PTZ) may be explained by a combination of previously described mechanisms. Overexpression of TEM and SHV beta-lactamases, possibly due to strong promoters or increased gene copy number (60–62), along with the presence of inhibitor-resistant variants such as TEM-33, IRT (59), or SHV (63), as well as the co-occurrence of multiple beta-lactamase genes, constitute important contributors to resistance to beta-lactam/beta-lactamase inhibitor (BL/BLI) combinations.

Additionally, inhibitor-resistant CTX-M variants such as CTX-M-255 and CTX-M-178 have been reported to exhibit reduced susceptibility to PTZ (64, 65). These enzymatic mechanisms are often accompanied by non-enzymatic factors such as porin loss (e.g., OmpC) (66) and efflux pump overexpression (67), both of which have also been associated with PTZ resistance.

In our study, PTZ resistance was primarily associated with the presence of the *bla*OXA-1 gene. In the first period, PTZ susceptibility was assessed in 69 isolates, of which 25 (36%) were resistant. Among these, *bla*OXA-1 was detected in 68% (17/25). In the second period, out of 54 isolates evaluated, 29 (54%) exhibited PTZ resistance, with *bla*OXA-1 identified in 48% of them (14/29).

The lower frequency of *bla*OXA-1 in the second period, despite the increased number of PTZ-resistant strains, may be partially explained by the overexpression of beta-lactamases such as SHV, whose prevalence increased during this period, as well as by the accumulation of multiple resistance determinants, likely due to the higher frequency of isolates harboring *bla*CTX-M-2 (Fig. 5).

Regarding the role of AmpC enzymes, previous studies have shown that these can contribute to PTZ resistance when overexpressed or combined with other mechanisms (68). However, in our setting, a recent national multicenter study conducted by the Antimicrobials Department of INEI—ANLIS “Dr. C. G. Malbrán” reported a *bla*AmpC prevalence of only 6% among 822 carbapenemase-producing Enterobacterales isolates in Argentina during 2023 (69). This low frequency suggests that AmpC was unlikely to have been a major contributor to PTZ resistance in our study.

To the best of our knowledge, this is the first study to investigate the prevalence of various beta-lactamase-encoding genes associated with the ESBL phenotype, alongside Class 1 integrons, in clinical samples from both ambulatory and hospitalized patients at a referral hospital in central-western Argentina. The study provides insights into how the prevalence of these genes has shifted in the context of social lockdown and the healthcare system overload experienced during the COVID-19 pandemic. In this context, the study demonstrates highly significant changes when comparing samples obtained before and after the pandemic. In our opinion, the most striking finding is that the prevalence of the ESBL phenotype in ambulatory samples almost doubled. This is particularly concerning, as the antibiotics commonly used to treat infections in ambulatory patients are becoming increasingly ineffective against an increasing number of cases, resulting in severe complications. For instance, the empirical use of broad-spectrum antibiotics without prior performance of antibiograms or sensitivity testing may lead to the administration of two or more antibiotics with no favorable outcomes, facilitating the selection of resistant microorganisms and increasing the risk of adverse effects for the patient (70). Additionally, delays in initiating appropriate treatment can result in serious health complications for affected patients.

Another notable finding was the rapid increase in the prevalence of the *bla*CTX-M-2 and *bla*SHV genes. For *bla*SHV, this phenomenon could be attributed to the dissemination of beta-lactamase inhibitor-resistant SHV variants capable of conferring an ESBL phenotype, or to the spread of strains and/or plasmids promoting high-level expression of SHV enzymes. This may be associated with the widespread use of amoxicillin/clavulanic acid, one of the most frequently prescribed antibiotics for empirical treatment of infections in Argentina. Regarding the blaCTX-M group 2, the significant increase in its prevalence during the second period remains unclear. It is possibly driven by the rapid dissemination of virulent bacteria harboring this gene and its horizontal transfer via conjugative plasmids (71).

Global evidence shows that although antimicrobial resistance is a widespread issue, local realities vary considerably (72, 73). This suggests that the clonal transmission of resistant bacteria between different regions occurs at a relatively slow pace and is more dependent on local practices than on geographical proximity. This presents a unique opportunity for each region to implement targeted actions aimed at delaying or mitigating the proliferation of non-endemic multidrug-resistant bacteria. To achieve this, it is essential to conduct regular surveillance studies for the early detection of virulent strains harboring a broad range of resistance genes, before they are clonally disseminated or resistance genes are horizontally transferred between bacteria.

We believe that at the local level, it is essential to allocate greater efforts and resources to strengthen healthcare infrastructure, enhance epidemiological surveillance, and train healthcare personnel. Additionally, it is crucial to politically empower specialists in the field, enabling them to actively participate in decision-making, in the development of tools and contingency plans, and their implementation in response to the potential emergence of super-resistant bacteria not typically found in the region.

On a global scale, it is imperative to invest in research focused not only on the discovery of new antibiotics but also on the decontamination of environments polluted with multidrug-resistant bacteria, both in hospital and non-hospital settings. Achieving this will require the exploration of innovative technologies, such as ecological niche competition, phage therapy, and other creative solutions.

Finally, studies related to antimicrobial resistance associated with ESBL production have recently lost relevance. However, 80 years after the discovery of the first antibiotic and in the absence of new antibiotics, we find to be deeply concerning the significant increase in the frequency of ESBL-producing bacteria among ambulatory patients, along with the presence of multiple BSBL and ESBL genes per bacterium. We consider it essential to highlight the importance of monitoring strains with the ESBL phenotype, particularly in ambulatory communities and in productive sectors. This is especially relevant as therapeutic options are increasingly limited, and the emergence of new antimicrobial agents is virtually non-existent. In this regard, it is crucial to understand that antibiotic resistance arises from a complex interaction of various factors and human practices (71, 74).

We acknowledge some limitations in our study. For instance, it would have been valuable to perform sequencing of at least some of the strains constituting the sample, with the aim of determining the resistome and mobilome. Additionally, the use of multi-locus sequence typing would have allowed for the assessment of the prevalence of potentially virulent strains within the ambulatory and hospitalized samples. This approach would have facilitated the comparison between the different time periods, enabling the determination of whether the observed trend toward greater homogenization in the prevalence of the analyzed genes in the post-pandemic period, between hospitalized and ambulatory samples, was due to clonal dispersion of hospital strains into the community or horizontal transfer mediated by mobile elements such as conjugative plasmids. However, due to resource limitations, this analysis was not feasible.
